# A hybrid blob-slice model for accurate and efficient detection of fluorescence labeled nuclei in 3D

**DOI:** 10.1186/1471-2105-11-580

**Published:** 2010-11-29

**Authors:** Anthony Santella, Zhuo Du, Sonja Nowotschin, Anna-Katerina Hadjantonakis, Zhirong Bao

**Affiliations:** 1 Developmental Biology, Sloan-Kettering Institute, 1275 York Avenue, New York, New York 10065, USA

## Abstract

**Background:**

To exploit the flood of data from advances in high throughput imaging of optically sectioned nuclei, image analysis methods need to correctly detect thousands of nuclei, ideally in real time. Variability in nuclear appearance and undersampled volumetric data make this a challenge.

**Results:**

We present a novel 3D nuclear identification method, which subdivides the problem, first segmenting nuclear slices within each 2D image plane, then using a shape model to assemble these slices into 3D nuclei. This hybrid 2D/3D approach allows accurate accounting for nuclear shape but exploits the clear 2D nuclear boundaries that are present in sectional slices to avoid the computational burden of fitting a complex shape model to volume data. When tested over *C. elegans*, *Drosophila*, zebrafish and mouse data, our method yielded 0 to 3.7% error, up to six times more accurate as well as being 30 times faster than published performances. We demonstrate our method's potential by reconstructing the morphogenesis of the *C. elegans *pharynx. This is an important and much studied developmental process that could not previously be followed at this single cell level of detail.

**Conclusions:**

Because our approach is specialized for the characteristics of optically sectioned nuclear images, it can achieve superior accuracy in significantly less time than other approaches. Both of these characteristics are necessary for practical analysis of overwhelmingly large data sets where processing must be scalable to hundreds of thousands of cells and where the time cost of manual error correction makes it impossible to use data with high error rates. Our approach is fast, accurate, available as open source software and its learned shape model is easy to retrain. As our pharynx development example shows, these characteristics make single cell analysis relatively easy and will enable novel experimental methods utilizing complex data sets.

## Background

Time-lapse imaging of optically sectioned nuclear images has provided an unprecedented opportunity to observe biological processes as they unfold in space and time. Using fluorescent proteins such as GFP to label nuclei, one can image the embryogenesis of diverse organisms such as *C. elegans *[1], *Drosophila*[2], zebrafish [3,4] and mouse [5,6] with single cell resolution over an extended period of time. Given sufficient temporal resolution, individual nuclei can be followed over time, providing a virtually contiguous record of proliferation, differentiation and morphogenesis [2,5,7-9]. However, exploiting this source of information is not trivial: such data sets can record thousands of cells over hundreds of frames, adding up to terabytes of files. The data becomes manageable only when tracks of the behavior of individual cells are generated from the raw images.

In response to this, a variety of computational techniques and software packages have been developed to aid in quantitative analysis of images [10-15]. The largest class of image analysis methods focuses on segmenting contiguous regions of pixels, implicitly detecting nuclear locations in the process. The simplest technique, thresholding image intensity, has been supplemented with image processing techniques like smoothing, adaptive thresholds [16,17], morphological operators, mode finding[18,19], watershed [20,21]and level set methods[22] to increase robustness in the face of noise, uneven contrast and touching nuclei. A second class of methods uses matched filters to detect the centers of nuclei. A predefined template of object appearance is compared to every location in an image and local maxima of similarity are assumed to be object centers [1,23,24].

The success of these methods is mixed. When nuclei are widely spaced, many methods perform well, with error rates of one percent or less [16,25,26]. However, as nuclear density increases and nuclei start to touch, which is typical of late embryonic development and adult tissues, error rates rise to from two up to more than ten percent [1,3,18,20,22]. Errors are caused by image characteristics that violate the rules or models underlying a detection method. These have their origin in both biology and the imaging process. Variation in nuclear fluorescence and shape is the primary biological complication. Uneven signal within a nucleus (Additional file 1, Figure S1a) can result in over segmentation, separate redundant detections of a nucleus on each mode of intensity. Elongated and irregular nuclear shapes can also contribute to over segmentation (Additional file 1, Figure S1c). Even if expression is uniform, each end or bump may independently match some computational definitions of a nucleus. Biology also contributes to under segmentation or missed nuclei. When nuclei are crowded, nuclei with weaker expression may be masked by brighter neighbors and remain undetected (see Additional file 1, Figure S1b for an illustration of a weaker nucleus).

Imaging distortions and resolution limitations further complicate nuclear detection. An optically sectioned sample yields a series of 2D cross sections, so that nuclei are recorded as slices through bright globular shapes against a dark background. Coordinates within the 2D planes are referred to as x and y while z refers to the direction orthogonal to the image planes. Optical and physical constraints make resolution along the z axis lower than that within the x,y plane. Typically, phototoxicity and image acquisition time also limit the number of optical sections, further reducing the z resolution. In turn, this limits the information available to resolve closely packed nuclei along the z axis (Additional file 1, Figure S1d illustrates two nuclei whose images merge along the z axis). Systematic distortions such as fading of signal with depth and stretching of fluorescent signal along the light path (Additional file 1, Figure S1e) further distort the picture, contributing to error.

All errors are problematic because the quality of information extracted during image analysis limits the kinds of biological questions that can be answered. A low percentage of error, say three or five percent, allows reliable assessment of statistical trends in the behaviors of a homogeneous group of cells, such as drug response in cell culture, or the shape and overall migration of a tissue. On the other hand, this level of accuracy is not always sufficient for detailed analysis of embryogenesis, where tracing the behavior of single cells is often necessary. Investigation of many critical developmental processes such as neural crest cell dispersion[27] or convergent extension [28] can be most effectively investigated with this kind of single cell record of development. Though desired information may be theoretically present in images, it is large-scale annotation of cell behavior that makes systematic investigation possible. Tracing the paths of cells as they move and divide demands virtually 100% accuracy, as a handful of misidentified cells per time point can lead to a thoroughly fragmented and scrambled lineage [1]. To make use of error filled data, biologists must spend a significant amount of time manually editing the automatically constructed result. Even for simple organisms with a few hundred cells, such as *C. elegans*, this has previously taken up to days [29]. In an organism like zebrafish this amount of editing would be an impossible task, with correction of even a sublineage of interest being weeks, or months, of effort. Errors quickly make human curation a bottleneck, undermining the premise of high throughput imaging. Often, when combined with time constraints, this inability to accurately find cells forces one to detour around a key question, missing an opportunity for a clean experimental design.

To address this challenge we present a detection and segmentation method that is accurate enough to allow high fidelity analysis over a variety of images while remaining fast enough to run in real time, making it practical for use with large data sets.

## Results

### Algorithm Design

**Figure 1:**
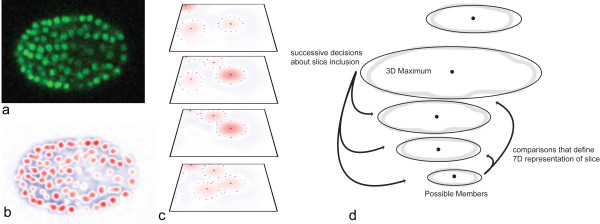
**Slice extraction and nuclear definition**. a. An x,y plane through C. elegans volume data at the ~350 cell stage. b. the corresponding slice through the 3D DoG filtered volume. c. Slices are segmented by casting out rays in search of a zero crossing. The 2D intensity maxima where rays originate are marked as black dots. Final end points of search rays are marked as blue dots. These points define a polygonal slice; multiple slices can be assembled together to yield a 3D nuclear boundary. d. Nuclear shape definition. The position, intensity, and size of each slice that might be part of a nucleus are measured relative to the nuclear center, and also relative to the closest slice between the possible member and the nuclear center. These measurements make up the 7D vector that represents a slice and nucleus center pairing. Actual nuclear extraction starts from the center and in turn considers the likelihood of each slice as an endpoint for the nucleus.

Given that image planes are widely spaced along the z axis, adjacent and similar voxels along the z axis are fairly likely to belong to separate nuclei, frustrating naive detection and segmentation methods. A shape model is necessary to guide segmentation, filling in boundary information that is not locally available in the image. Our method views nuclei as a collection of slices and uses a shape model that consists of expectations about slice size, brightness and location relative to other slices (See Figure 1 for a graphical overview of this process from image data to segmented nucleus).

This basic shape model is sufficient because nuclei have relatively simple, largely convex shapes. Pixel level segmentation is done within 2D image planes, a shape model is not necessary because resolution is high (Figure 1c). In contrast, the final definition of 3D nuclear extent, which must be done under the constraint of limited resolution along the z axis, is based on pre-segmented slices, allowing computationally efficient use of a model of nuclear shape (Figure 1d). This approach avoids the impossibly high computational cost of fitting complex shape models, like the active shape models [30] commonly used to segment noisy volumetric data, to hundreds of thousands of nuclei.

**Figure 2:**
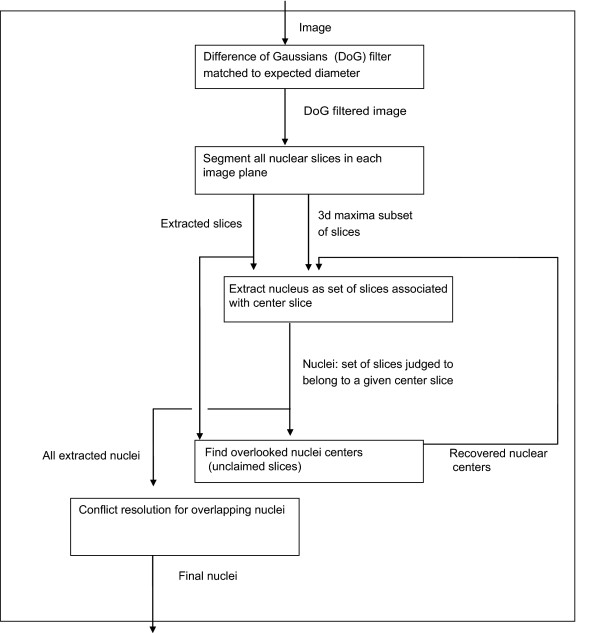
**Flowchart overview of the algorithm**. Boxes represent major elements of the algorithm and arrows the flow of data between them.

Figure 2 provides the flow of our algorithm, which starts with finding and segmenting all nuclear slices, and then uses local 3D maxima as seeds for extracting nuclei as a set of slices. Unclaimed slices give rise to new nuclear seeds, and competing claims on the same slices are resolved by choosing between merging the overlapping nuclei or giving slices to the nucleus with the stronger claim. Below, each step of the algorithm is detailed with a subheading corresponding to each box in the flow diagram. Implementation details are provided in Additional File 1: Supplemental Methods and Figures, section 1.

### Image filtering

**Figure 3:**
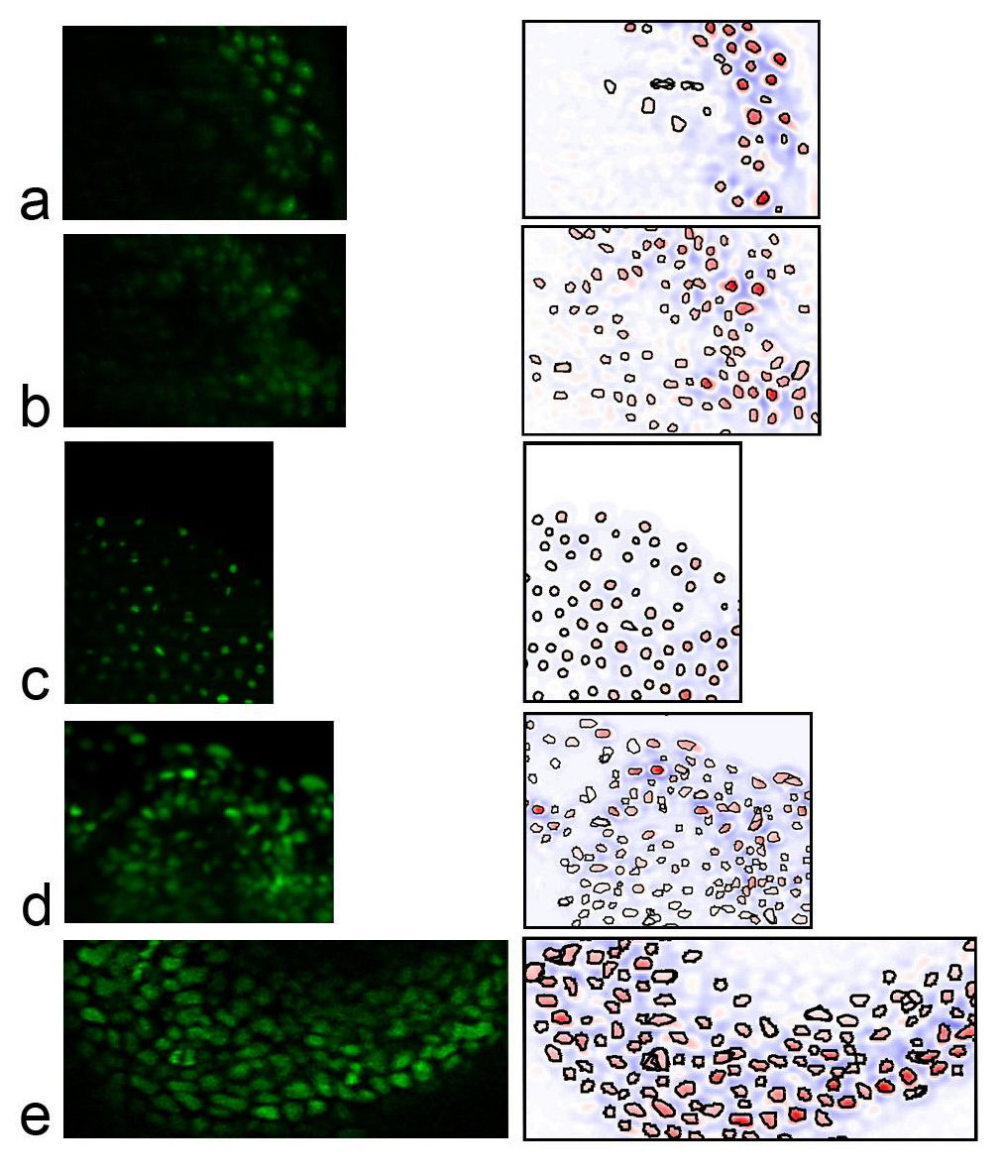
**Test Data**. A representative plane of test sets and corresponding slice segmentation (see Figure 1 for *C. elegans *example plane). a. early *Drosophila *(stage 8, ~4 hpf) b. late *Drosophila *(stage 11, ~7 hpf) c. early zebrafish (~3 hpf) d. late zebrafish (~18 hpf) e. mouse ~E7.75

Our approach begins with image filtering to find an initial set of seed locations for nuclei. Volume data is filtered with a 3D Difference of Gaussian's (DoG) filter [31], which can be viewed as a matched filter representing a blurred sphere against a dark background. As in typical use of such filters, 3D maxima of intensity in the filtered image serve as candidate nuclear centers for further analysis. At the same time, the filtering process also highlights information about the individual nuclear slices: 2D local maxima within each image plane represent the centers of nuclear slices, and the edges of nuclei are highlighted as zero crossings of intensity (Figures 1b and 3). See Additional File 1: Supplemental Methods section 1.1 for more details.

### Slice Segmentation

Based on the filtered volume data, nuclear slices are extracted as polygonal regions. For every 2D intensity maxima above a base threshold a 2D shape representing that slice through the nucleus is segmented. Following our goal of reducing computational complexity, 2D segmentation is reduced to a set of 1D boundary detection problems. Sixteen evenly spaced rays are sent out from the 2D maxima. These rays terminate when they encounter a zero-crossings (illustrated in Figure 1c). Ray endpoints are then post processed to discard rays that are unusually longer or shorter than their neighbours. These final ray endpoints define a polygonal segmentation boundary that can capture detailed shape, but is computationally very cheap to compute. See Additional File 1: Supplemental Methods section 1.3 for more details.

### Nuclear Extraction

We then combine extracted slices and the subset of these slices corresponding to 3D maxima to segment nuclei. The slice corresponding to each 3D maximum attempts to claim slices above and below it based on a trained probabilistic model of nuclear shape. The underlying shape model is a set of 7 dimensional gaussian distributions, generated using labeled training data, and representing the expected properties of slices within a nucleus and of 'distractor' slices originating from other nearby nuclei. Dimensions of this distribution include the relative size, intensity and position of slices in relation to the center slice and the closest intervening slice (shown in Figure 1d). Training the model is simple, a superset of slices that might be part of the nucleus is generated automatically and extra slices are deleted to create a corrected result (see readme in Additional File 2: source code for detailed instructions). This model is used in a simple maximum likelihood classifier that assesses whether each nearby slice along the imaging axis is more likely to belong to the nucleus or another nearby 'distractor' nucleus. This shape model, though simple, is flexible enough to capture different levels of shape variation, nuclear separation, and optical distortion, making it adaptable to different organisms and microscopy techniques. See Additional File 1: Supplemental Methods section 1.4-1.6 for more details.

### Finding Overlooked Nuclei

When crowded, nuclei with weaker fluorescence intensity are often overshadowed by brighter neighbours, and so are not 3D maxima. Conceptually, we can imagine masking the signal from known detected nuclei. When this is done dimmer nuclei become local maxima. The segmentation of all nuclear signal into the unit of slices provides a practical way to achieve this. All slices claimed by at least one nucleus are marked as accounted for and the remainder examined. In any 3D spherical neighbourhood where multiple unclaimed slices exist, an overlooked nuclear center is seeded at the locally brightest slice. This works because the shape model is accurate enough to prevent the brighter, initially detected nuclei from claiming the slices corresponding to these undiscovered dimmer nuclei. These nuclei are extracted from the full set of slices (including claimed ones) subject to the same nuclear shape model above, and this is repeated iteratively until no unclaimed clusters of slices remain. See Additional File 1: Supplemental Methods section 1.7 for more details.

### Conflict Resolution

False positives come largely from multiple detections of the same nucleus caused by variation of intensity within the nucleus. False positives from noise are rare because of the strong smoothing provided by filtering. Most false positive cases are revealed by significantly overlapping claims on slices from multiple (equivalent) nuclei. These cases are judged by a conflict resolution step. Whenever two extracted nuclei both claim a slice, two possibilities are considered, merging the two nuclei, and splitting their overlap between them. These two configurations are scored against the shape model and the option with the best shape score is picked. Merging is scored by using the shape model to calculate the total score of all slices in a nucleus formed from the union of all slices in both nuclei, assuming its center to be the slice closest to the geometric middle of the merged set of slices. The second possibility, splitting, is scored by assigning each slice to the nucleus with the strongest claim on it, subject to the constraint of each nucleus being contiguous and adding up the total of the two nuclei's claim on their slices. See Additional File 1: Supplemental Methods section 1.8 for more details.

### Image analysis software

Matlab source of the image analysis software is available as Additional File 2: source code and is distributed under the GNU GPL. The source will be actively maintained; the most recent version is available for download at sourceforge (starrynite.sourceforge.net under the file subheading blob-slice cell detection).

Though the algorithm contains a significant number of parameters, the set of key parameters is relatively small. It is typically possible to get good results by setting only two intuitive parameters: the diameter of the nucleus in the first frame, used to set the filter size, and the noise threshold used to discard filtered maxima corresponding to image noise. A full catalogue of all parameters (Additional Table S3 and S4) and advice on tuning them for new images is provided in Additional File 1, section 2.1. An example parameter file along with a script and instructions for retraining the 3D shape mode is provided in Additional File 2: source code.

### Evaluation of Accuracy

We applied our method to a diverse set of test data, encompassing both confocal and light sheet microscopy, and sampling a range of metazoan model organisms. Data sets include *in toto C. elegans *using laser scanning confocal microscopy [1], *in toto Drosophila *using DLSM-SI [32], *in toto *zebrafish using DLSM [3] and a partial mouse embryo using laser scanning confocal microscopy (see Figure 3 for representative slices and Additional file 1, Table S1 for image resolution and other details). For *C. elegans*, we analyzed 280 time points covering roughly five hours of development and ranging from the four-cell stage to about 500 cells. For each of the other data sets, sub volumes containing 200-400 cells were selected, due to the time constraint of gathering the ground truth by manual identification of nuclei when the guidance provided by an invariant lineage is not available. For *Drosophila *and zebrafish data, which cover an extended period of development, an early and a late developmental stage were tested. We analyzed *Drosophila *stage eight and eleven (approximately four and seven hours post fertilization (hpf) and with ~6,000 and 14,000 total cells respectively) and the zebrafish late "1K cell" and "14-19 somites" stages (approximately three and eighteen hpf and with ~1,500 and 15,000 total cell respectively). The mouse embryo is analyzed at the late headfold (embryonic day (E) 7.75) stage with all non-axial mesodermal nuclei labeled. When selecting the sub volumes, we chose regions with above average difficulties in the respective data in terms of nuclear density, variation of nuclear shape, size and intensity. Parameters were tuned on one time point and then tested on three successive time points for the same test sub volume.

**Figure 4:**
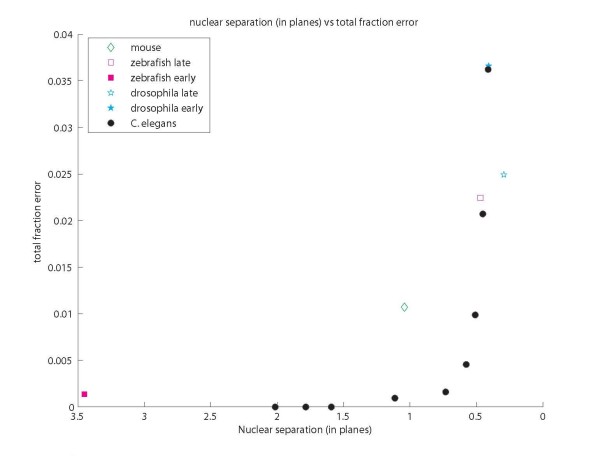
**Nuclear separation as a predictor of performance**. Nuclear separation is calculated as the average distance between the computed boundary of a nucleus and the boundary of its nearest neighbor, based on the bounding circle of the largest slice. This distance is expressed in units of slice spacing, the distance between successive z planes. Averages are displayed; error variability between data sets was typically 1-2 mistakes.

All error rates were calculated based on ground truths created by human correction of all discernable detection errors in the computed result. Segmentation accuracy was not considered, as our primary goal is localization of nuclei, segmentation is largely a way to increase the accuracy of that localization. However, some segmentation results are shown in Figure 3 and appear to be of good quality. For *C. elegans *the detection ground truth can be considered perfect, as the invariant lineage provides a guide in resolving any temporary image ambiguity. For all other data sets the ground truth was generated by examining the volume data slice by slice several times, deleting multiple detections and marking overlooked nuclei. The ground truth was automatically matched against the original result and deviations logged as errors. Computed results and ground truths are available as Additional File 3. *C. elegans *image data is available on request because of its large size; images for other organisms are included as Additional Files 4, 5, 6 and 7 This additional data is release under the GNU GPL. Average error rates range from near zero for early zebrafish and *C. elegans *to around 3 to 3.7% in *Drosophila *and late *C. elegans *(Figure 4). These error rates are about two to six fold lower when compared to previous approaches. For early development in *C. elegans*, our method achieves 0.25% error around the 180-cell stage, compared to 1.98% reported for data of equivalent quality with a mode finding approach [18]. Our approach also compares favorably with 0.43% error reported using a graph cut segmentation method [26] on another highly similar dataset. Performance on later developmental stages was not reported for either of these methods. For late *C. elegans *development, our method achieves ~0.5% between the 180- to 350-cell stage (the ninth and second to last round of cell division) and ~3% afterwards (350- to ~500-cell stage). Our previous matched filter method [1] yielded ~3% and 12% error respectively at these stages using the identical test data and ground truth. Error on the zebrafish data set is a third or less (~2.5% vs ~10% at the 14-19 somites stage) than that of the adaptive thresholding method originally used on the same data set [3]. While our assessment is based on a portion of the whole volume used in prior error analysis the sub volume is one of the most crowded areas in the image.

We further analyzed how different components of our algorithm contribute to the final accuracy of the results. We analyzed one of the data sets that gave the highest error rate, namely the late stage *C. elegans *embryo from about 350 to 500 cells. The breakdown in Table 1 illustrates the contribution of the shape model, both in finding missing nuclei in the form of unclaimed slices, and in merging redundant overlapping segmentations of the same nucleus. These steps reduce both false negatives and false positives by about a half from the initial 3D blob detection.

**Table 1:** Error rates at each stage of the algorithm for the 350- to 500-cell C. elegans embryo.

	Initial DoG 3D maxima detection	Overlooked nuclei added	Final error after overlap resolution
False Negatives (%)	4.6	1.7	1.8

False Positives (%)	1.8	2.8	1.3

Total error (%)	6.4	4.5	3.1

Error rates compared to ground truth for the partial result available at each stage of the algorithm.

In analyzing error rates we found that accuracy correlates well with the separation of nuclei in z across organisms and imaging methods (see graph in Figure 4). Error is close to zero when the average separation is greater than or equal to one plane. This means that, on average, gaps between nuclei are captured by one or more planes. Error rises sharply when the average separation is less than half a plane. In contrast, sampled slices per nucleus, an intuitive measure of visibility, does not predict accuracy (Additional Figure S2). These results emphasize that, because the gap between nuclei is typically much smaller than nuclear size, resolvability (rather than visibility) of nuclei bounds performance. This suggests that for quantitative analysis z sampling should be carefully tuned in response to the spacing between nuclei. In spite of this, our experience suggests that experimenters tend to be less attentive to this parameter, compared for example to laser intensity, likely because image quality within a plane is more perceptible. The mouse data is an exception to this trend, z sampling is more than adequate but error is higher than expected because the x,y resolution is unusually low (imposed by the technical requirements of keeping the embryo healthy during imaging). This causes occasional segmentation errors and somewhat higher than expected error (Figure 3 and Additional file 1, Table S1 illustrate the mouse data and give its resolution). A qualitative sense of analysis results can be gained from Additional files 8 and 9, 3D reconstruction movies of the mouse and Zebrafish embryos generated from the unedited output of our system.

### Computational cost

To assess running times and memory loads, we tested our method on a whole volume of each data set on a 2.13 GHz Intel core2 PC using a lightly optimized single threaded Matlab implementation (Additional file 1, Table S2). The runtime consists of several components, with disk access and image filtering scaling with image size while slice and nuclear extraction scale with the number of nuclei present. In larger images computational time is strongly dominated by image filtering, which contributes to 83% of runtime for the zebrafish dataset. For the *C. elegans*, *Drosophila *and mouse data, the processing speed is about 3 seconds per megapixel of image data. For the zebrafish data, the speed is reduced to approximately 6 seconds per megapixel. This is likely due to memory management inefficiencies resulting from the need to divide the image filtering into multiple parts that fit within addressable memory on a 32bit architecture.

It is worth noting that, even in this unoptimized implementation on limited hardware, our algorithm is fast and efficient enough for real time analysis of all data sets but zebrafish. As the core image filtering is highly parallelizable and memory management seems to take a large toll in our tests, a parallelized implementation on a fairly typical multicore 64bit workstation with sufficient memory would be qualitatively faster. This should allow the processing of larger volumes such as the zebrafish data in real time with a few CPUs. The computational cost of our method compares favourably with previous work. Our method takes ~23 seconds per volume to process *C. elegans *data, well below the data sampling rate of one volume per minute. In contrast, mode finding [18] takes twice as long, on a volume less than half the x,y resolution using comparable hardware. Our detection combined with our previous tracking approach [1], takes about 37 seconds per volume to detect and track through the 180 cell stage, compared to up to 20 minutes per volume at the 180 cell stage for combined tracking and segmentation with a graph cut approach [26]. Our method is also efficient in comparison with previous zebrafish analysis methods both of which would require around two hours [3,22], to process a volume of the size that our method can segment in approximately 20 minutes.

### 3D reconstruction of pharynx development

We demonstrate the potential of our algorithm by analyzing the organogenesis of the pharynx in *C. elegans*. The pharynx is a prime model for organogenesis and has been widely studied. However, a detailed single cell level record of its formation has never been made. This is because of the image analysis challenge presented by small cells in a crowded configuration during later embryogenesis. With our previous cell detection method [1] high error made this analysis impractical. More accurate detection opens the door not only for this record of wild type development, but also for novel experimental investigations of late pharynx morphogenesis at the single cell level.

The pharynx is a feeding apparatus that ingests and grinds bacteria. It is made of 80 cells, derived from different lineages and including multiple tissues such as muscles, neurons and glands [33,34] [WormAtlas.org]. This complex set of tissues with a small set of cells has made the pharynx a powerful model to study organogenesis. Genetic and functional genomic analysis have identified the key signaling pathways, master regulators and the molecular cascade underlying pharyngeal development, leading to a substantial understanding of how the cell lineage generates the particular set of 80 differentiated cells (see Mango, [35] for a detailed review). However, as in most models of complex organogenesis, how the differentiated cells give rise to the structure of a functioning organ is poorly understood.

**Figure 5:**
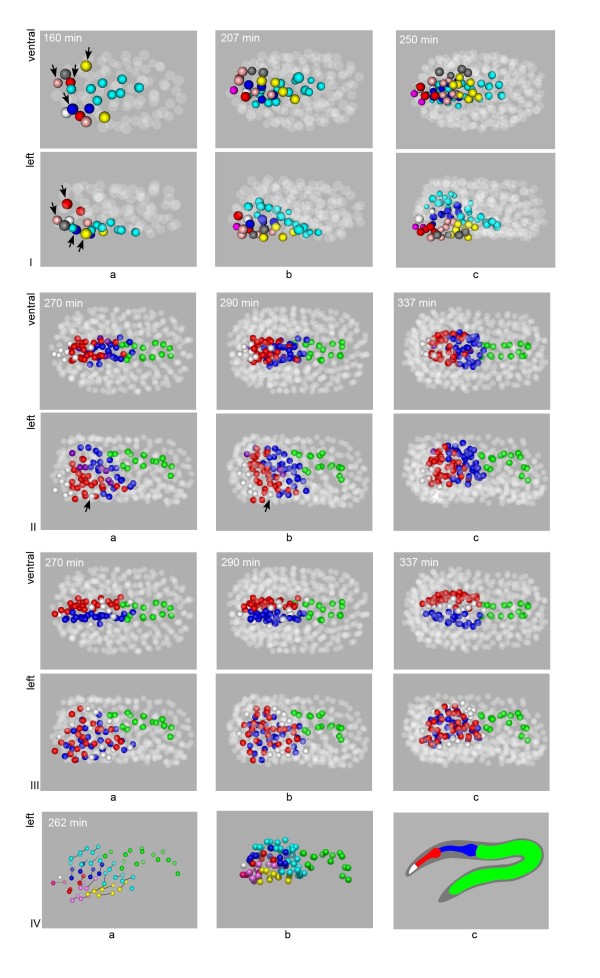
**Reconstruction of *C. elegans *pharynx development**. **I**. Assembly of the primordium. MSaa and MSpa lineages are in cyan. On the right side ABaraaap (and then its anterior daughter) is in red, ABarapaa in pink, ABarapap in yellow and ABaraapp in blue. On the left side, the symmetric sublineages are shown in the same colors but are marked by arrows, with ABalpaap (and then its anterior daughter) in red, ABalpaaa in pink, ABalpapp in yellow and ABaraapa in blue. White in frame a represents ABaraaaa, which gives rise to two L/R symmetric sublineages (in magenta in frame b and c) as well as a pair of cells one of which undergoes apoptosis and the other of which forms the third fold of symmetry for part of that sublineage (white in frame b and c). Grey represents a non-pharyngeal precursor, ABalpapa which interrupts the left side group at birth (frame a) but is excluded during subsequent development. For all frames in this figure, the non highlighted cells are shown as semi-transparent spheres. In frame a, at time 160, left-right symmetric precursor cells have been born but are not symmetric in their layout. Note the midline marked by the two rows of MS/cyan cells. MS cells have just started to enter the inside of the embryo. The blue cell that is part of the left side is born on the right side of the midline but will cross over to join the other left side cells. In frame b, time 207, the AB pharynx cells have moved to the midline to cover the MS cells. The blue cell of the left group has crossed the midline to assume a symmetrical position as its right counterpart. However, the pink cells of the left group are still disconnected from the yellow cells compared to the right side. The grey non-pharyngeal cells are now excluded from the primordium. In frame c, time 250, the left and right AB groups are fully assembled and symmetrical. **II**. The inflation of the primordium. To illustrate the topological mapping of the primordium to the mature pharynx, cells are colored as follows: white for buccal cavity, red for the corpus/anterior lobe, blue for the posterior lobe and purple for precursors whose descendents contribute to both lobes. The E/gut cells are shown in green for context. Frame a shows the primordium prior to inflation, where cells are arranged in two flat sheets that are left-right symmetric. In Frame b the sheets have begun to round slightly. In c they have rearranged to create a rounded shape, and the ventral MS portion of the pharynx moved anterior to the E cells. **III**. The emergence of threefold symmetry. Pharyngeal right side terminal cells (and their precursors) are in blue, those on the left are in red. Terminal cells and precursors are white if they, or their descendents, have no L/R counterpart. These cells make up the third component of the final threefold lumen symmetry. **IV**. Frame a shows the correspondence between pharynx cells whose lineages are annotated as left right symmetric with a line. A left view, angled slightly posterior-dorsal y, highlights the consistent alignment. Frame b, the position of cells at ~340 min pfc. Frames a and b use the same color scheme as in I with the addition of the E/gut cells in green. Frame c shows the final configuration of the pharynx colored as in II.

We have reconstructed pharyngeal development up to the stage where structures corresponding to the parts of the fully formed pharynx can be visually identified in the embryo (~340 minutes post first cell cleavage, pfc). Visualized in 3D, early morphogenesis of the pharynx appears to involve two distinct stages. During the first stage, pharyngeal precursor cells are recruited from discrete regions of the embryo to form a coherent structure with an overall left-right symmetry (Figure 5I, Additional file 10: Movie 3). Pharyngeal cells are derived from the AB and MS lineages, with the MS cells born in a contiguous structure and the AB cells assembled piecemeal. In the MS lineage, pharyngeal precursors are born in two rows, one on the left side and one on the right (cyan in Figure 5I). The two rows are born next to each other and the midline corresponds to the future midline of the organ, around which the AB cells assemble. The AB cells can be further divided into two groups. The right side group is derived from the ABara sublineage. Cells in this group (red, pink, yellow and blue in Figure 5I) are born next to each other (Figure 5I frame a) and maintain their relative positions as they move towards the midline to meet the left side group (Figure 5I frames b and c). In contrast, the corresponding cells in the left side group (sharing colors with their left side fate counterparts in Figure 5I but marked with arrows) are born isolated and migrate towards each other to assemble a mirror image of the right side group (Figure 5I frames b and c). In the meantime, the pharyngeal precursors move from the ventral surface to the inside of the embryo. This process starts with the MS cells at around 160 minutes pfc (Figure 5I frame a). The AB cells first move on the ventral surface towards the midline (see above) to cover the MS cells (Figure 5I frame b) before following them inside (Figure 5I frame c). This stage of morphogenesis, which we term the assembly stage, ends at ~250 minutes pfc. The end result is a contiguous primordium consisting of two flat sheets of cells and an overall left-right symmetry. This is highlighted in Figure 5IV frame a, which marks the correspondences between pharyngeal cells from symmetric lineages.

During the second stage of morphogenesis, which we term the inflation stage, the flat, two-sheet structure swells, similar to the inflation of a balloon, to create a rounded structure. Figure 5II and Additional file 11, Movie 4 illustrate the process with a color scheme that shows the mapping of the primordium to the mature pharynx (Figure 5II frame b) [34][WormAtlas.org]. Interestingly, shortly before the inflation starts (Figure 5II frame a), the primordium is aligned with the anterior-posterior axis between the anterior end of the embryo and the intestine (green in Figure 5II). From the ventral view, this configuration is similar to that of the mature pharynx, creating a false impression of the mapping to the final structure. As the left view shows, the longitudinal axis of the digestive tract is curved and deviates from the long axis of the whole embryo, that is, the anterior-posterior axis. The inflation starts around 280 minutes pfc (Figure 5II frame b) and becomes prominent by 320 minutes. It starts from the middle of the primordium with no obvious bias towards the future anterior or posterior lobe. The relative position of cells largely remains constant during the inflation. A dramatic exception is the e2V cell (marked with an arrow in Figure 5II), which moves anteriorly from the middle of the primordium to join the other epithelial cells that make the buccal cavity. We also followed the symmetry of the primordium over time. The mature pharynx shows a threefold rotational symmetry, while the cell lineage and the primordium show largely bilateral symmetry. As Sulston pointed out, "the third symmetry element arises [in the lineage] by piecemeal recruitment of cells", and the placement of these cells in the lineage does not show any apparent logic or regularity [33]. As shown in Figure 5III and Additional file 12: Movie 5, these cells (white) are born at or near the midline along the length the primordium, on both the dorsal and the ventral sides. Thus, the logic controlling which cells are recruited for the 3D symmetric structure is apparently spatial. Additional file 13:Movie 6 illustrates the final 3D configuration of cells colored by lineage origin, anterior/posterior and left/right fate, allowing their systematic comparison.

More detailed temporal and spatial information is available in movies corresponding to different coloring schemes in Figure 5 (Additional files 10, 11, 12 and 13: Movies 3 to 6). As individual cells within the organ can be analyzed based on our accurate nuclear identification method, morphogenic behaviours can be dissected at single-cell resolution using mutants, gene expression mapping and other approaches. Such studies will ultimately extend the molecular cascade of pharyngeal development from differentiation to morphogenesis, and pave the way for a comprehensive understanding of organogenesis.

## Discussion and Conclusion

Our method is a reliable tool for nuclear identification that, by respecting the structure of the underlying image data, achieves robust and fast nuclear detection and segmentation. Three key ideas underlie the strength of our design. First, we separate the problem of 3D nuclear segmentation into 2D slice segmentation and 3D slice grouping. We can achieve reliable 2D segmentation using simple methods because of the high resolution within image planes. We can then efficiently solve the hard problem of 3D segmentation by grouping the relatively small number of slices in the image (typically 3 to 4 per nucleus at late developmental stages). Second, we employ a trainable, probabilistic shape model based on slices, which allows us to consider the variability of nuclear shape and intensity within an easily manageable framework. Third, we use the 3D maxima generated by the DoG filter to guide nuclear segmentation. As 3D maxima typically provide >95% accuracy in nuclear detection, they provide a powerful guide for a greedy slice grouping approach. The strategy of segmenting individual slices of volume data is not unknown [22,36,37], but our method is unique in its computational strategy and in being general, fully automatic and capable of good results in the crowded images typical of late embryogenesis. The algorithm remains efficient and fast enough for a typical computer to achieve real-time analysis of *in vivo *imaging of metazoan embryogenesis, in models ranging from *C. elegans *to mouse.

Aside from accuracy and speed, our method has the advantages of easy adaptability and an extensible modular framework. Retraining the probabilistic nuclear model for new images provides increased flexibility with minimal effort. Since slices can typically be segmented with >95% accuracy after setting only 2 simple base parameters, manual labeling involves only pruning a list of nearby potential slices. No hand tracing of image regions is necessary. Detailed tuning and training procedures are given in Additional File 7 section 2. Apart from the adaptability of the probabilistic model, our method has the added flexibility of a modular algorithmic design. Each sub task is largely independent of the others and can be replaced by other methods independently of the general strategy. For example, if very differently scaled or shaped nuclei were a concern, the DoG filter could be replaced by a (more computationally expensive) battery of oriented or multi-scale filters while leaving the rest of the framework untouched. Additional specializations can also be added at different stages to address particular concerns, such as false detections outside the boundary of an embryo or adaptation to fading signal.

Currently, our method still produces 2-3% error in the more challenging cases of late embryogenesis. The vast majority of remaining false negatives are crowded nuclei for which secondary recovery failed because a brighter neighbor not only prevented its detection as a 3D maximum but also mistakenly claimed all of its slices. Similarly, the majority of remaining false positives are nuclei split into upper and lower halves along the z axis. These were detected twice but did not claim each other's slices sufficiently to be merged. This suggests that the classifier for slice inclusion is likely the weakest link in our system and could be improved, especially for the elongated nuclei with varying spatial orientation that are frequently seen in the drosophila and mouse embryo. However, at least half of these error cases are ambiguous to the eye, and require examination of adjoining time points to determine if they represent one or two nuclei. This suggests performance of our algorithm may be close to the bound set by information in a single image, and large future improvements may be more easily achieved through improvements to imaging and use of temporal tracking.

As our analysis on the relationship between the error rates and nuclear separation (Figure 4) suggests, the most direct imaging improvement for detection in most situations would be an increase in z resolution. Additional resolution in the x,y plane or improved signal to noise ratio are always useful, but if x,y resolution is already sufficient for segmentation this will not significantly reduce error and might be counterproductive if, for example, it results in increased phototoxicity due to greater magnification or laser power. Our results provide a practical guide for optimizing imaging parameters: ensure a z sample spacing of at least the separation between nuclei. Though acquisition speed and other constraints will not always allow a sufficient z resolution, the curve in Figure 4 allows these factors to be traded off against error in an informed manner. Furthermore, because nuclear separation is a universal metric for fluorescence labeled nuclear images, it is useful in comparing image analysis techniques across different image data. For example, this metric highlights the counterintuitive fact that the ~3 hpf zebrafish embryo with over 1500 cells is about as easy to analyze as the twenty four cell *C. elegans *embryo.

## Methods

### Imaging protocols

Zebrafish [3] and *Drosophila*[32] data are published data sets captured with DLSM and DLSM-SI microscopy techniques respectively. Imaging resolution and temporal sampling information for these and all other data sets are detailed in Additional file 1, Table S2 and Table S1.

*C. elegans *4-D confocal images were recorded with a Zeiss Axio Observer.Z1 with 491-nm laser at a temporal resolution of one minute for embryos between the two cell and ~ 540 cell stages (320 minutes). To normalize for loss of fluorescence in lower focal planes and increase in fluorescence later in development, adjustments were made to laser power (ranging from five to thirty percent) and exposure time (from eighty five ms to 120 ms) according to slice and developmental stages.

Mouse embryos expressing a H2B-GFP reporter specifically within non-axial mesoderm where dissected and imaged as detailed in [38].

### Pharynx Development Analysis

Nuclei were detected using our method, then tracked using StarryNite[1] with errors corrected manually using AceTree [29]. Nuclei were followed until approximately 340 minutes pfc. Pharyngeal cells were identified by their lineage identity, which, given the invariant cell lineage of *C. elegans*, equates with fate[33].

Image analysis algorithm details are available in Additional File 1, section 1.

## Authors' contributions

AS designed and implemented the algorithms, drafted the majority of the manuscript and edited detection results. ZD optimized and performed *C. elegans *imaging and drafted the related portions of the manuscript. SN and AH optimized and performed mouse imaging and drafted the related portions of the manuscript. ZB participated in the design of the algorithm and revised the manuscript. All authors read and approved the final manuscript.

## Supplementary Material

Additional file 1 **Additional Figures and Methods**. Details of image analysis algorithm, supplemental figures and instructions for tuning parameters. Click here for file

Additional file 2 **Additional Source Code**. Source code for the detection algorithm, retraining the shape model, and an example parameter file corresponding to the early zebrafish data. Zip file contains Matlab source and related files. Click here for file

Additional file 3 **Additional Data and Ground Truth**. Computed nuclear positions and corrected ground truths for all example data contributing to Figure 4. Zip file contains data in Acetree format comma separated value text files. An explanation of the Acetree file format is included in readme.txt. Click here for file

Additional file 4 **Additional Images Zebrafish - late, Drosophila, Mouse**. The sub-volumes of image data used in experiments. Each Matlab dat file contains a 3d matrix 'stack' of intensity values with dimensions ordered y,x,z. Click here for file

Additional files 5 **Additional Early Zebrafish image data**. The sub-volumes of image data used in experiments. Matlab format array data as above; because these are larger each is included as a separate additional file. Click here for file

Additional files 6 **Additional Early Zebrafish image data**. The sub-volumes of image data used in experiments. Matlab format array data as above; because these are larger each is included as a separate additional file. Click here for file

Additional files 7 **Additional Early Zebrafish image data**. The sub-volumes of image data used in experiments. Matlab format array data as above; because these are larger each is included as a separate additional file. Click here for file

Additional file 8 **Movie 1: Mouse Reconstruction**. 3D movie illustrating somites in the mouse embryo based on our system's analysis of a full volume of mouse test data. Click here for file

Additional file 9 **Movie 2: Zebrafish Reconstruction**. 3D movie showing the internal structure of a Zebrafish embryo based on our system's analysis of a full volume of Zebrafish test data. Click here for file

Additional file 10 **Movie 3: Early Pharynx Development**. 3D animation of the assembly stage of pharyngeal development based on detection results. See legend of Figure 5I for an explanation of the coloring scheme. Click here for file

Additional file 11 **Movie 4: Precursors of the corpus and posterior bulb**. 3D animation showing side by side left and ventral views of the embryo during the inflation stage of pharyngeal development. See legend of Figure 5II for coloring scheme. The movie illustrates the detailed reshaping of the pharynx from 197 through 337 minutes. In the later half of the assembly stage (197 to 250 minute), the two sheets expand in size through division. During the final round of synchronized divisions between ~277 and 307 the pharynx contracts along the AP axis rounding slightly. On the completion of divisions this structure then inflates to form a roundish structure[33] prior to its eventual elongating and spitting into two chambers (not shown). This ballooning is apparent from time 317 onward and occurs at the same time as the ventral MS pharynx cells move anteriorly toward the main mass of the pharynx. The movie also highlights the mouth precursors being born relatively distant from their final positions and converging near their final location. Click here for file

Additional file 12 **Movie 5: Establishment of 3 fold symmetry**. 3D animation of side by side ventral and anterior views of the embryo during the inflation stage of pharyngeal development. See legend of Figure 5III for coloring scheme. Click here for file

Additional file 13 **Movie 6: Structure of pharynx and contributions of different sublineages**. 3D rotation of the pharynx at time 337 makes the 3D structure clear and allows comparison of the final time points in the coloring schemes of movies 1, 2 and 3. Click here for file
