# Supplementary material for: A hybrid blob-slice model for accurate and efficient detection of fluorescence labeled nuclei in 3D
Source: BMC Bioinformatics. 2010 Nov 29;11:580. doi: 10.1186/1471-2105-11-580 (PMC3008706; doi:10.1186/1471-2105-11-580)
Supplement: Additional file 1 — Additional Figures and Methods. Details of image analysis algorithm, supplemental figures and instructions for tuning parameters. [file 1471-2105-11-580-S1.DOC]

**Additional Tables, Figures and Methods**

**Additional** Table 1 Image sampling resolution for each test data set.

|  | *C. elegans* | *Drosophila* | Zebrafish | Mouse |
| --- | --- | --- | --- | --- |
| X,Y res (µm) | .254 | .37 | 1 | 1.1 |
| Z res (µm) | 1 | 3.7 | 5.18 | 1.5 |
| Voxel aspect ratio | 3.937 | 10 | 5.18 | 1.36 |
| Nuclear diameter (µm) | ~5-3 | ~4.6-3.3 | ~20-8.6 | ~9.5 |
| Nuclear separation (µm) | ~2-.4 | ~1.5-1.0 | ~18-2.4 | ~1.5 |

**Additional Table 2** **Computational load of processing a full image volume.**

|  | *C. elegans* | *Drosophila* | Zebrafish | Mouse |
| --- | --- | --- | --- | --- |
| Volume dimensions (pixels) | 512x512x30  (7.86 mega pixels) | 1490x636x60 (56.9 mega pixels) | 1016x1054x193 (206 mega pixels) | 512x512x146 (38.3 mega pixels) |
| Temporal sampling (minutes between samples) | 1 | 3 | 1.5 | 15 |
| Number of detected cells | 180 | 3922 | 1533 | 2397 |
| Runtime (minutes) | .38 | 2.37 | 20.8 | 2.14 |
| Maximum memory usage (Megabytes) | 438 | 1,091 | 2,211 | 1,051 |

**Additional Table 3 Parameter settings used for all test data sets.** Where multiple settings were used at different stages these are comma separated in time order within each cell. The *C. elegans* parameters are broken into blocks staged by # of cells 0-24, 25-79, 80-180, 181-250, 251-350 and 351 onward. In the other data sets multiple numbers represent the late and early stage time points.

|  | ***C. elegans*** | ***Drosophila*** | **Mouse** | **Zebrafish** |
| --- | --- | --- | --- | --- |
| **Image filtering** |  |  |  |  |
| Initial Nuclear Diameter (pixels) | 40 | 13,10 | 7 | 20,7 |
| DoG Filter size (nuclear diameters) | 1 | 1.1 | 1 | 1.75,1 |
| Noise threshold (filtered pixel intensity) | 8,10,14,16,21,21 | 11 | 5 | 10,8 |
| **Slice segmentation** |  |  |  |  |
| *MinDrop* ( fraction intensity reduction) | .3 | .6 | .5 | .5 |
| *MaxRayChange*  (fraction ray length increase) | 1.5 | 2.5,2.25 | 2.5 | 2 |
| *MinRayChange* (fraction ray length decrease) | .333 | .333 | .333 | .333 |
| **Nuclear extraction** |  |  |  |  |
| *-logOddsThreshold* (minimum negative logodds for inclusion) | 100,100,15,8,4,3 | 100 | 8 | 100,5 |
| **Conflict Resolution** |  |  |  |  |
| *SplitThreshold* (threshold ratio btw total logodds) | 1,1,.5,.5,.3,1 | 1 | 1 | 1 |
| *MinSplitThreshold* (total logodds score) | 100,100,20,19,17,5 | 100 | 0 | 10,5 |
| *MinMergeThreshold* (total logodds score) | -300,-200,-100,-35,-15,-20 | -200,-100 | -30 | -30,-20 |
| *AspectRatioThreshold* (ratio between nuclear height and diameter) | 1.6,2,1.6,1.3,1.1,.55 | 1.6 | .5 | .75,.5 |
| *DistanceThreshold (in nuclear diameters)* | .8,.6,.6,.5,.4,.4 | .8 | .5 | .75,.5 |

**Additional Table 4 Parameter summary and advice.**

| **Image filtering** |  |
| --- | --- |
| Initial Nuclear Diameter (pixels) | At the first time point this parameter needs to be manually set to the size of the nuclei. For elongated nuclei it should be set a little higher than the short axis. |
| DoG Filter size (nuclear diameters) | 1 is usually a good value. If nuclei are very separated and strongly multi modal GFP is causing FP this can be raised to smooth more, in that case minDrop typically needs to be raised also to compensate for this blurring. |
| Noise threshold (filtered pixel intensity) | Threshold for a significantly bright slice. This should be set a little lower than the brightest filtered 3D maxima corresponding to a nucleus. |
| **Slice segmentation** |  |
| *MinDrop* ( fraction reduction) | The drop in value from the maximum which is deemed ‘close enough’ to a zero crossing. .3 is a reasonable default (a 70% drop). if the image appears blurry it should be set a bit higher to prevent overestimation of diameter and hence over smoothing. |
| *MaxRayChange*  (max fraction increase) | The maximum allowable difference between 2 adjacent ray lengths. 1.5 is a good default. More elongated nuclei need a higher value, but this carries some risk of merging crowded adjacent nuclei. |
| *MinRayChange* (min fraction decrease) | The minimum allowable difference between adjacent slices. It is not typically necessary to adjust this, |
| **Nuclear extraction** |  |
| *-logOddsThreshold* (minimum negative logodds for inclusion) | This is the primary parameter influencing 3D nuclear extraction and iterative discovery of overlooked nuclei. When nuclei are well separated it will have relatively little effect, but should be set relatively high to avoid splitting nuclei into multiple fragments during extraction and to ensure nuclei initially detected twice are merged (which will not happen if their slice sets do not overlap). When images are relatively crowded a low single digit number is typically best, representing a slight bias to claim unlikely looking slices, but not so high as to cause the claiming of entire separate nuclei. |
| **Conflict Resolution** |  |
| *SplitThreshold* (threshold ratio btw total logodds) | How much better the split score needs to be than the merge score before overlapping nuclei are split. 1 is a good default value, it can be decreased to bias toward merging if too many FP are present, but the gain is somewhat minimal for low throughput applications, being fractions of a percent of error. |
| *MinSplitThreshold* (total logodds score) | Threshold on split score below which things will be merged even if their merge score is not great as long as it is better than MinMergeThreshold below. A small single digit number is a universally safe starting point. Again this parameter is less critical. |
| *MinMergeThreshold* (total logodds score) | Really bad merge score threshold above which things can be merged if their split score is also terrible. With MinsplitThreshold above these 2 parameters define an unbounded upper left rectangular gating region corresponding to malformed nuclei that are always merged with their overlapping neighbor. |
| *AspectRatioThreshold* (ratio between nuclear height and diameter) | Aspect ration between height (in z) and diameter. If 2 nuclei when merged have an aspect ratio below this cutoff they are merged regardless of merge/split scores. Given the presence of optical distortion that makes nuclei look stretched in z a relatively high number for this e.g. 1.5 is preferable in uncrowded images, while in crowded images nuclei may appear much shorter than their diameter requiring a threshold below 1. |
| *DistanceThreshold (in nuclear diameters)* | Distance between their centers at which nuclei with overlapping slice sets are merged regardless of other scores. In crowded and undersampled images detected centers are often much closer to each other in z than would appear possible given physical constraints. This requires relatively low thresholds .5 (meaning the center of one nucleus should not sit within the radial boundary of the other) is a typically safe value. In uncrowded images this can be increased because nuclei are more distant and overlaps are much more likely to correspond to multiple detections of one nucleus. |

**Additional Figure 1** **Illustrations of image properties that complicate image analysis schemes.** a. A zebrafish nucleus showing uneven, multi modal GFP distribution within the nucleus (each mode is marked with an arrow). b. Variation of nuclear intensity in *C. elegans*, the dimmer nucleus marked with an arrow (each of the three central nuclei are cross-sectioned at their brightest middle plane). c. Mouse nuclei showing a range of highly elongated and irregular shapes. d. x-z cross section of three C. *elegans* nuclei illustrating that though resolution within a slice (horizontally in this image) may be sufficient that nuclear boundaries are always clear, the boundary between nuclei along the z axis (marked with an arrow) can be lost. e. x-z cross section of *C.* *elegans* nucleus illustrating lower sampling in the imaging direction as well as optical distortion which elongates the nucleus in the z direction.


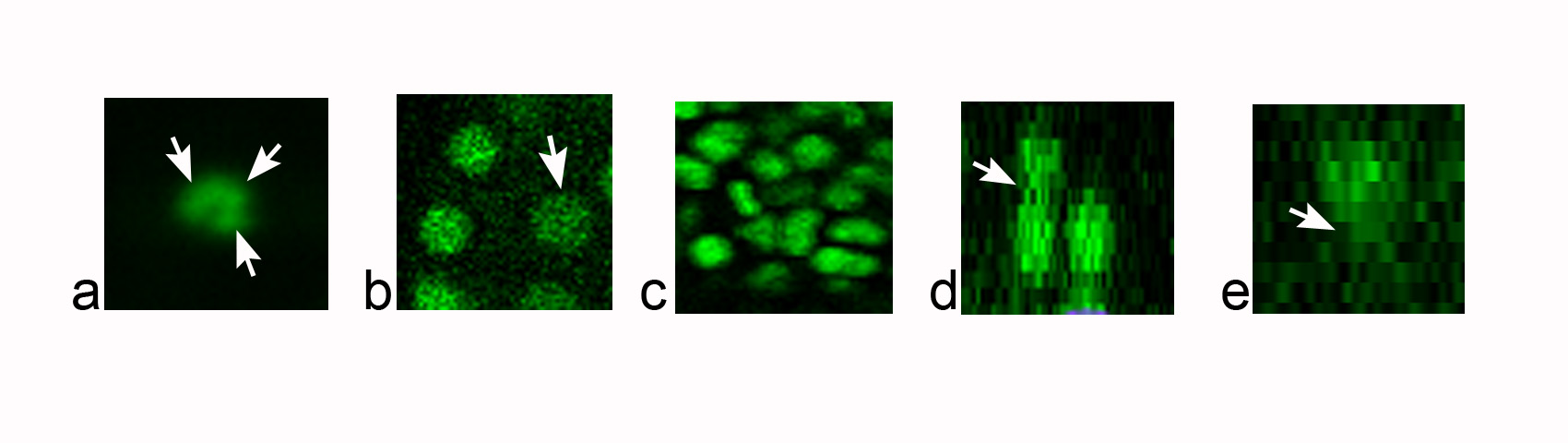


**Additional Figure 2 Nuclear diameter does not predict performance**. Unlike nuclear separation, nuclear size does not appear to correlate smoothly with error. Though it is intuitive to think of the size of an object as related to the ease of detecting it, this is not the limiting factor in typical images, where distances between nuclei are much smaller than nuclear size. Even if a nucleus intersects with a relatively large number of planes, (three for late stage *C. elegans*) poor accuracy can result if nuclei merge together.


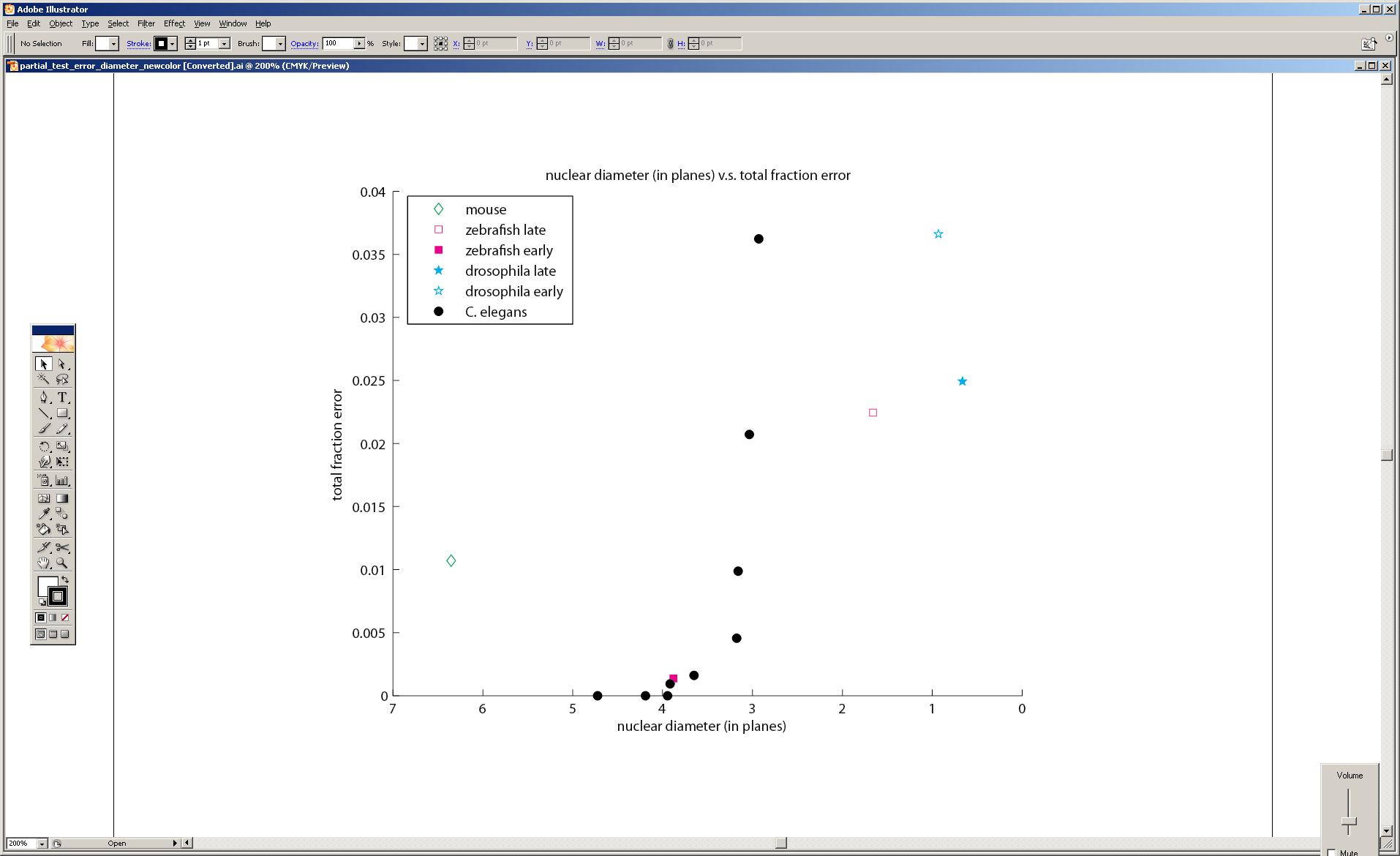


**Additional Methods**

**Section 1: Algorithm details**

**1.1 Image filtering**

Image data is filtered with a 3D Difference of Gaussians filter sized to match expected nuclear diameter. Specifically, the smaller Gaussian filter of the pair has a sigma such that its full width at half height is one half the expected nuclear diameter. The larger filter is a fixed factor of 1.6 times the size of the smaller. At the first time point this size is set manually; this is a key input parameter of the system. At successive times expected size is estimated using the average of extracted diameters at the previous time point. Because image data is not sampled uniformly in each dimension, the size of the filter in each dimension is adjusted based on sampling to preserve symmetry in physical 3D space. For example, if each voxel is 1µm x 1µm x 2µm a filter with size s x s x s/2 is used. Filtering is implemented with a FFT. The FFT of large volumes and the corresponding DoG filter could not fit in addressable memory on a 32 bit architecture, so this computation was broken into smaller sized tiles which were fitted together.

**1.2 Slice and Nuclear Center Definition**

Seeds for nuclear slices are defined as local intensity maxima within each filtered x, y imaging plane, that is, voxels whose intensity is greater than or equal to all eight connected nearest neighbors within the plane. Initial nuclear seeds are the subset of slice seeds which are also greater than or equal to the additional eighteen voxels in its twenty six connected three dimensional neighborhood. All maxima below a low noise threshold are discarded from both slice and nuclear center sets. This threshold is set manually for each image type and major developmental stage. Given this threshold, false positives on the whole result from fluorescence variation within nuclei and not noise. If fading with plane depth were problematic it would be simple to modulate this threshold by a measured signal loss function.

**1.3 Slice segmentation**

Slices through nuclei are segmented by sending out sixteen radially arranged rays from the intensity maxima. This is illustrated in Figure 2. These rays travel until they near a zero crossing, reaching a point with a value less than *MinDrop* times the value at the maxima or alternatively fail to find such a point within a maximal distance of 1.5 times the expected diameter. If *MinDrop*=0 (appropriate if images are very crisp and high contrast) this finds the actual zero crossing in the DoG image which typically corresponds to the boundary of bright areas [28]. Higher values of *MinDrop* yield the isocontour around the maxima corresponding to a specified fraction of the maximal value (in *C. elegans* *MinDrop* =.3 a 70% drop from the intensity maxima value). This compensates for blurring due to diffusion which would otherwise cause exaggerated nuclear size. If a positive minima is passed in this process it is separately recorded.

The sixteen ray end points (if found) are filtered for validity by removing rays that are unusually long or short compared to their neighbors. Unusual length suggests that these rays have stopped short on some minor feature, or have overshot because of a missing section of zero crossing and instead rest on the further edge of an adjoining nucleus. Starting with the median length ray, which is assumed to be valid and taken as a reference, this ray’s length is compared to that of the next clockwise ray for which an end point is defined. If this next ray is greater than *MaxRayChange* times the length of the reference ray, or shorter than *MinRayChange* times the reference ray, it is marked as invalid. If the test ray is invalid, the current reference ray is retained and the next neighbor is considered. If the test ray is valid, it becomes the reference ray, and the process is repeated. If a zero crossing is not found within the cutoff distance or is labeled as too long and a minima was found this minima is used in its place. This simple smoothness check has typically been sufficient to achieve a useful shape approximation. If a more accurate boundary of very elongated or irregular shapes is necessary a second order smoothness check could be substituted. Our approach is motivated by speed and ease of implementation, more robust 2D segmentation methods like active contour or shape models could be used to segment slices if image quality were poorer or higher fidelity boundaries were needed and run time was unconstrained.

A bounding circle is then defined for each slice. We define the ray coverage of the slice as the number of rays which have a valid distance associated with them. If ray coverage is greater than thirteen (the overwhelmingly typical case, ~93% of the time in the latest stage *C. elegans* data analyzed) then the centroid of the polygon represented by the end points of the valid rays is calculated and this is the center of the circle. The radius of the circle is set equal to the eightieth percentile of the distances of the polygon boundary points from the centroid. If ray coverage is less than or equal to thirteen the polygon represented by the rays is judged unreliable. The center of the circle remains at the initial intensity maxima, and diameter is set the average expected diameter. At this point all of the significant modes of GFP signal in the data have been segmented into slices, and these remain to be grouped into nuclei.

**1.4 Slice feature definition**

A nucleus can be assembled from slices by searching up and down from a nuclear center for a contiguous set of slices that cohere in position and intensity. If nuclei were spherical and well separated this would be trivial; slices would be aligned in z along a small set of planes, decrease in intensity and size from the center, and nearby nuclei would be separated by a plane with no nearby slices. This ideal is not so far from the truth, even at late stages this is the case the vast majority of the time. In order to quantify the variability found, we represent the appearance and location of a slice relative to its proposed nucleus as a point in a seven dimensional space. The dimensions of this space are:

1. The distance in the z dimension between the slice and the nuclear center (the number of planes lying between them).
2. The distance in the x,y dimensions of the slice’s centroid and that of the nuclear center (the distance between the projections of the two points into the same image slice plane).
3. The distance in the x,y dimensions of the slice’s centroid from that of the closest slice on the next plane in the direction of center plane (which is redundant with 2. in the special case where the slice adjoins the center) .
4. The difference of slice bounding circle size with the nuclear center.
5. The difference of slice bounding circle size with the closest slice on the next plane in the direction of center plane.
6. The difference in maximal intensity between the slice and the nuclear center.
7. The difference in maximal intensity between the current slice and the closest slice on the next plane in the direction of center plane.

All distances are normalized by the center slice bounding circle diameter; all intensities are normalized by the center slice maximal intensity to reduce variance caused by absolute intensity and nuclear size. These measures provide a compact, but relatively complete picture of the appearance and location of a slice relative to a proposed nucleus it might be part of. Our challenge is then to characterize nuclear shape by modeling the natural variability in these measurements in order to determine whether or not a slice belongs with a particular nucleus. This numerical model includes the variability found within a nucleus and also the change encountered when crossing over the nucleus’ boundary into another nucleus.

# 1.5 Nuclear model training

To use this definition of a slice in extraction, we first need to train a model of the variability we expect to see in images. To generate a labeled training set, first we extract a superset of possible slices around a nucleus. A cylinder with a diameter equal to the center slice’s bounding circle and a length three times the diameter is sufficiently large to capture a superset of slices that are part of a nucleus. For a set of a few hundred training nuclei, a corrected segmentation was hand generated by deleting all incorrect slices from this set, this took a couple hours.

Using this labeled data set, a simple model based on a set of Gaussian probability density functions (PDFs) is trained. Four distributions are trained. Two of the distributions represent slices that are part of nuclei, one above, one below the center slice within the image stack. The other two distributions represent distracter slices found within each half of the cylinder that are not part of the nucleus. The first two slices encountered in each direction that are not part of a nucleus are used to create the outside slice distribution. These closest distracters are more representative of ambiguous cases than all distractor slices (most of which are far away). Separate slice and distractor distributions are trained for the part of the nucleus above and below the center slice because we observe that in confocal microscopy (as can be seen in Figure 1e) distortions tend to elongate the image of nuclei preferentially in the direction facing away from the light source. Our learned model means that if this distortion is not present the two distributions will be equivalent. Means and full covariance matrices are calculated for each set of points.

On an intuitive level, this model captures shape by characterizing the variability in size, position and intensity that one expects to see within slices that belong to a nucleus, as well as the degree of divergence that one expects to see when crossing into another nucleus. This representation is very simple, and computationally cheap to work with, but it is expressive enough to capture the major sources of variability that complicate nuclear extraction. Through the z distance this model can capture optical distortion along the z axis, for example, the fact that in many cases the images of nuclei extend into planes more than a radius away from their center. The x,y distance measures can capture drift in slice position relative to the center due to shape variation and the influence of signal from nearby nuclei. The slice size measures capture variability in slice size throughout the nucleus. Size is typically decreasing (as the brightest point is often also the largest point) but this is variable in some images due to nonspherical shape and GFP non-uniformity.

**1.6 Nuclear Extraction**

As in collecting training data, a first step to extraction is finding a superset of all slices which might be part of a nucleus. Starting at each center slice, a contiguous (containing at least one slice on every plane) stack of slices is extracted around the center. This is done by moving up and down in the z stack from the center slice. If any slices on the next plane have a centroid that lies within the bounding circle of the center slice, all these are included in the potential slice set, and the next plane is examined. If no slices are found or if the next plane is further than one and a half diameters away from the center this search stops. This search yields all slices whose centers fall in a one by three diameter cylinder of space centered on the center slice, truncating the cylinder if a plane is encountered that contains no slices (this would correspond to a nucleus cleanly separated from its neighbors).

To decide if a potential slice is included in a nucleus we use our trained distributions in a simple maximum likelihood classifier. For each candidate slice in each nucleus the 7D feature vector is calculated. The model PDFs for the appropriate half of the nucleus are evaluated at the point corresponding to each slice. Using the probability densities as relative probabilities, we calculate the log odds of slice membership in the nucleus as log(pdf_in(slice_vector)/pdf_out(slice_vector)) . The higher this value is, the more likely the slice is to be part of the nucleus, with 50/50 chance of membership (a position directly on the decision surface) corresponding to log odds zero. Any classification technique (e.g. Support Vector Machines ) could be used in place of this approach to characterize the training data and assign membership strengths. Other classifiers might be more robust to training data outliers or could capture more complicated conditional patterns for defining inclusion. This might be helpful in particularly crowded, difficult images, but this simple approach has worked well in our experience.

Because a nucleus should be a contiguous region of space we cannot directly use the classifier result. The range of candidate slices actually included as part of a center can be defined by starting at the center and separately working outwards toward both ends. All candidate slices on a plane with log odds scores > *logOddsThreshold* are included. If no slice has a score better than the threshold or if there are no candidate slices on the next plane the process stops. *logoddsThreshold* is set to a small negative value, adding a slight bias toward including slices. This is a tunable parameter that can be changed to avoid the need to train a new distribution for every developmental stage. At early stages where nuclei are well separated there should be very few slices in the candidate set which are not part of the nucleus (even at late stages the majority of nuclei do not have any incorrect slices in their candidate sets). Setting the threshold to a high negative number captures this fact without explicitly retraining the slice distributions for each spatial/temporal region. Note that this could equivalently be phrased as a ‘prior probability’ of inclusion used to bias the odds ratio, but this provides no advantage as long as the value remains a user defined parameter. It should be noted that this assignment of slices to nuclei is not exclusive. A slice can be determined to be a member of multiple nuclei, these conflicts of ownership are useful in detecting false multiple detections, and are discussed in the section Conflict Resolution below.

**1.7 Finding Overlooked Nuclei**

When detected nuclei have claimed all likely member slices, some slices are left unclaimed. Some of these are mistakenly unclaimed fragments of detected nuclei; others are nuclei that have been overlooked entirely. To identify overlooked nuclei we first remove all claimed slices. Remaining slices are filtered to identify possible nuclear centers by examining a sphere one and a half times the expected nuclear diameter around each unclaimed slice centroid. If nothing else is found in this sphere then the slice is considered an isolated fragment and discarded. If a brighter slice is found within the sphere the slice is suppressed by the brighter one and ignored. Locally brightest slices remaining are considered overlooked centers and are extracted as above. This process is iterated till no further potential centers exist.

**1.8 Conflict Resolution**

After all nuclei have been extracted, all overlaps (situations in which a slice appears in the slice set of multiple nuclei) are examined. All unique pairs of nuclei that overlap with each other are generated and each pair is independently considered. For each pair a decision is made between merging the two nuclei together, and splitting their overlapping region. Several measures are used to judge whether to merge or split each pair:

- *MergeScore* is the sum of slice log odds scores when the union of the two nuclei’s claimed slice sets is rescored with the geometric midpoint of the set chosen as the new nuclear center. This captures how much the union of the two slice sets matches our model of a nucleus.
- *SplitScore* is the sum of the log odds scores that result from splitting the union of slices between the two nuclei. Each nucleus gets the slices it has the stronger claim on but this division is made such that the nuclear ranges are contiguous and their summed log odds score is maximal. Each nucleus claims the portion of its own range that is further from the other nucleus center. Each plane that lies between the two centers is then tested as a dividing point. The sum of the slice scores relative to the center claiming them is added up for each dividing point. The maximal sum found is *SplitScore*. The corresponding dividing point will be used to divide the two nuclei if they are split. This measure captures how well the nuclei score when their overlap is resolved by splitting.
- *MergedAspectRatio* is calculated as the distance between the furthest slices in the union of slices from both nuclei divided by the diameter of the largest slices bounding circle.
- *CenterDistance* is the distance between the centers of the two overlapping nuclei.

Overlapping pairs are merged if:

1. *MergeScore*>*SplitScore*SplitThreshold*
    *SplitThreshold* is a parameter (usually set to one) which biases the decision toward merging or splitting. When this rule is true it suggests that the merged slice set better fits the nucleus model than the two individual nuclei.
2. *SplitScore*<*MinSplitThreshold* and *MergeScore*>*MinMergeThreshold*
   This rule defines a rectangular decision area, unbounded in two directions, in which nucleus pairs that score poorly for merging but also score very poorly for splitting are merged anyway. Nuclei that score very badly when split, so, as long as their union does not look absolutely terrible, are probably odd fragments that do not match the model well and should be merged.
3. *MergedAspectRatio* <*AspectRatioThreshold*
   If the aspect ratio of the nuclei when merged is still unusually small it is presumed that at least one of then is a mis-extracted fragment and they are merged regardless of score.
4. *CenterDistance* <*DistanceThreshold*
   *DistanceThreshold* (typically one half the expected diameter) is a distance at which centers are considered so close they are assumed to be the same nucleus. This is a typical post processing test in matched filter detection approaches, but here is applied only to nuclei with overlapping slice sets. This prevents the removal of even very closely packed nuclei if the slice inclusion model can distinguish them.

After all decisions have been made, merge decisions that include a nucleus or any nucleus it merges with are collected and single merged nucleus is created containing the union of all slices.

**1.9 Bottom detection**

If an embryo is imaged *in toto* it may be useful to calculate a bottom for the embryo, a plane below which all detections are the result of noise and diffused signal, and are discarded. Bottom detection was implemented as a post processing step. The number of nuclei found on each plane over all time points was tallied. The bottom was selected as the highest plane with less than a quarter of the maximum number of nuclei, combined over all times, found on any plane. This was useful in the confocal *C.* *elegans* data due to strong diffusion of signal at the bottom of the embryo. This simple definition of a flat bottom plane was appropriate here because the embryo is compressed between the coverslip and the glass slide.

**Section 2: Parameter Tuning and Training Instructions**

**2.1 Parameter Tuning**

If tuning parameters for new data the C. elegans parameters which correspond to a similar level of separation among nuclei are a good neutral place to start, unless the data strongly resembles one of the other data sets. Minimally two key things need to be set in the parameter file

1. firsttimestepdiam, the diameter of the nucleus in the first frame in x,y pixels
2. parameters.intensitythreshold The noise threshold which can be most efficiently set by processing one time point, plotting a histogram of the maxima values and picking a threshold that divides maxima from noise and from nuclei. Or, set the parameter to a reasonable small number and test the result visually; it is correct when there are no obvious nuclei sitting on noise in the ‘black’ areas of the image.

If nuclei are elongated max ray change might be made higher.

If nuclei are blurry minDrop can be raised to make computed diameters smaller. If this is not done it will result in diameters that are too large which over multiple time points may get progressively worse with many nuclei ultimately being missing.

Beyond this, parameters are related to the tradeoff between discovering overlooked nuclei, and merging nuclei that have been detected multiple times, in images with well separated nuclei other parameters will have relatively little influence on result quality.

*–logOddsThreshold(* parameters.rangethreshold) Is the most important and intuitive of parameters controlling merging and the discovery of overlooked nuclei. Increasing it will allow the inclusion of increasingly bad scored slices into nuclei. This will result in fewer overlooked nuclei being added , and potentially in a greater number of merge events being considered (because more slice inclusion will create more slice overlap), but this second effect is not strong.

All other merging related parameters can be nudged up and down with parameters in Supplemental Table 3 above giving a guide to their useful range, but are less essential to the algorithm and could be safely left at the relatively conservative parameters for 350 cell stage *C elegans.*  Merging parameters are most easily quantitatively tuned against a ground truth, to assess the result of a change. Absent this, it is probably most useful to nudge the aspect ratio threshold, and distance threshold up when too many multiple detections are found, and nudge them down if too many crowded nuclei appear to be missing.

**Section 2.2 Training**

Typically, for roughly spherical nuclei the included shape model is sufficient for reasonable quality results. Extraction using the shape model can be made more or less conservative by changing the –logOddsThreshold parameter to encourage the claiming of more or less slices by each nuclear seed. The default model for example was trained on 9^th^ round of cell divisions *C. elegans* nuclei and the –logOddsThreshold parameter increased at earlier stages (see Supplemental Table 3 ) to make it more liberal in its inclusion of slices when there is less opportunity for confusion because nuclei are well separated.

Training the shape model involves first running the algorithm on a volume of data to segment all slices, then hand editing the set of slices making up each nucleus, and calculating values based on this corrected result.

Specific, step by step, instructions for generating a new training set and creating a corresponding shape model are included in the Readme.doc file within the source code distribution.
