# Supplementary material for: A hybrid blob-slice model for accurate and efficient detection of fluorescence labeled nuclei in 3D
Source: BMC Bioinformatics. 2010 Nov 29;11:580. doi: 10.1186/1471-2105-11-580 (PMC3008706; doi:10.1186/1471-2105-11-580)
Supplement: Additional file 2 — Additional Source Code. Source code for the detection algorithm, retraining the shape model, and an example parameter file corresponding to the early zebrafish data. Zip file contains Matlab source and related files. [file 1471-2105-11-580-S2.ZIP › distribution_code/Readme.doc]

**Blob-Slice Nuclear Detection**

Blob-Slice Nuclear Detection is prototype open source software distributed under the GNU GPL. It was written in the Bao Lab, Sloan Kettering Institute, NY, NY. Contact Anthony Santella [santella@mskcc.org](mailto:santella@mskcc.org) with questions.

**Running the program**

commandlineDriver.m or commandLineDriver.exe are the wrappers for the core cell detection program

**Input**

Three or four input arguments are needed, these are:

1. *parameterfile*: the full path location of the parameter file testdata_parameterfile.txt provides an example parameter file appropriate for analysis of the example c elegans data.
2. *embryodir*: the full path of the directory containing image files
3. *embryonumber*: the prefix of the image file name
   if the filetype (specified in the parameter file, see file comments for details) is a saved matlab image array then files are presumed to be named *embyronumber*_####.mat and contain a 3d array variable *stack* containing the image data with dimensions 3 being z. Also supported are side by side red/green lsm files named *embyronumber*_GR#_B1.lsm and individual slice tiffs named *embyronumber*-t###-p##.tif. Appropriate switches need to be set in the parameter file for each data type.
4. *suffix* is an optional string that will be appended to the name to all output keeping one labeling of a data set separate from another of the same data set.

e.g. commandLineDriver('c:/test/parameters.txt',’c:/test/images/','rootimagename','test_run');

or, more specifically, assuming the provided test data is placed in 'G:\My Documents\supplemental_material\’:

commandLineDriver('G:\My Documents\supplemental_material\testdata_parameterfile.txt','g:\My Documents\supplemental_material\testdata\','ZD_BV24_JournalV_1_s1','test_run');

**Output**

Output takes 3 major forms

1. directory *embryodir*/matlabnuclei/ is created containing separate nuclear positions and nuclear diameter files for each time point processed (this can be suppressed from the parameter file).
2. *embryodir*/*embryonumber*_fullmatlabresult.mat Contains the final internal state of the nuclear detection code including all parameter settings. The important results data structure is *esequence* a cell array of a structure for each time point processed. Besides fields *finalpoints* and *finaldiameters* which match the data saved to the text files in 1 this structure contains *finalmaximas* the filtered intensity for each nucleus center as well as records of the nuclei found at each intermediate stage of the algorithm and extra data used in calculations like cell counts and diameter estimates.
3. *embryodir*/matlabnuclei/images/tif is created containing image data spit into tiff slices readable by Acetree. This output can be suppressed from the parameter file

**Viewing Output**

To view results in matlab you need to reload the original image data and display computed nuclei superimposed. To do this for the a time point of the example data, run these lines with the computed result in the matlab workspace.

timepoint=280;

X=im2double(((loadCellStackMetamorph([embryodir,embryonumber],timepoint,1,slices,[0,0,0,0],zeropadding))));

points= esequence{timepoint-start_time}.finalpoints;

dummymatch=linspace(1,size(points,1),size(points,1));

displayData(dummymatch,dummymatch, points,points ,X)

**Parameter file**

Detailed comments about the elements of the parameter file are included in comments within the file. The file is organized into three sections.

1. Main program control parameters for image resolution, size and time points to analyze
2. Algorithm parameters, with the simplest parameters most likely to require adjustment at the top. In general the noise threshold shouls be set first. Staged parameters included are for *C. elegans* test data
3. System configuration parameters. These control the functioning of the program itself, the type of image data it expects to be handed, optional controls of output, and a variety of controls for specialized usage.

**Training new slice distributions**

Images that differ significantly from the sample data in crowding or sampling or microscope distortions might benefit from retraining the slice inclusion model. This is done with the script distributionTrainingCode.m.

To retrain:

1. Run the program with the existing distributions on a set of training data you want to use.
2. Load the .mat file that is output by the program into matlab
3. Pick a time point to use in training and set in on the comment line time=277;
4. Cut and paste into matlab the section of commented code in distributionTraining_code.m labeled: RUN THIS FIRST. (this may take a few minutes to run) Note that the subdirectory NIFTI_20080408 must be in the matlab path in order to display the image data.
5. Using the image slice browser which comes up edit a few hundred of the nuclei slice sets in variable corrected_centers. This is done by opening the variable in the matlab variable editor and deleting the rows corresponding to slices that should not be in the nucleus.
6. As you edit nuclei add their index to the array ‘good=[];’. This specifies which nuclei in corrected_centers have been corrected and can be used in training.
7. Run the main script distributionTrainingCode.m it will calculate new distributions, and output their accuracy in classifying the training data.
8. Save the variables allbadrm, allbadlm, allgoodrm, allgoodlm, allgoodlc, allgoodrc, allbadlc, allbadrc to a .mat file.
9. Change the parameter to point to this new distribution file.
